# Properties of Electrospun Nanofibers of Multi-Block Copolymers of [Poly-ε-caprolactone-*b*-poly(tetrahydrofuran-*co*-ε-caprolactone)]*_m_* Synthesized by Janus Polymerization

**DOI:** 10.3390/polym9110559

**Published:** 2017-10-27

**Authors:** Muhammad Ijaz Shah, Zhening Yang, Yao Li, Liming Jiang, Jun Ling

**Affiliations:** MOE Key Laboratory of Macromolecular Synthesis and Functionalization, Department of Polymer Science and Engineering, Zhejiang University, Hangzhou 310027, China; muhammadijazshas@yahoo.com (M.I.S.); yznfly@zju.edu.cn (Z.Y.); 21429011@zju.edu.cn (Y.L.); cejlm@zju.edu.cn (L.J.)

**Keywords:** electrospinning, nanofibers, multi-block copolymers, elastomer, Janus polymerization

## Abstract

Novel biodegradable multiblock copolymers of [PCL-*b*-P(THF-*co*-CL)]*_m_* with PCL fractions of 53.3 and 88.4 wt % were prepared by Janus polymerization of ε-caprolactone (CL) and tetrahydrofuran (THF). Their electrospun mats were obtained with optimized parameters containing bead-free nanofibers whose diameters were between 290 and 520 nm. The mechanical properties of the nanofiber scaffolds were measured showing the tensile strength and strain at break of 8–10 MPa and 123–161%, respectively. Annealing improved their mechanical properties and their tensile strength and strain at break of the samples increased to 10–13 MPa and 267–338%, respectively. Due to the porous structure and crystallization in nanoscale confinement, the mechanical properties of the nanofiber scaffolds appeared as plastics, rather than as the elastomers observed in bulk thermal-molded film.

## 1. Introduction

Poly(ε-caprolactone) (PCL) is a commonly used biocompatible and biodegradable polymer, having extensive applications in medical fields [1,2,3,4]. PCL is semi-crystalline, brittle, and hydrophobic. Its degradation via hydrolysis is appropriate for long-term drug carriage [5,6]. An auspicious material for biomedical applications can be achieved by merging the favorable, essential properties of the polymer PCL with the distinctive characteristics of a nanofibrous structure [7,8,9,10]. The degradation and mechanical properties can be amended by copolymerizing or blending with other polymers [5]. Tetrahydrofuran (THF) has been polymerized and studied over the last 50 years. Both the synthesis and properties of THF-based polymers were widely reported in the literature [11]. THF can only undergo cationic ring-opening polymerization (ROP) to produce polytetrahydrofuran (PTHF), which is widely used as the soft segments in thermoplastic elastomers such as polyurethanes and polyesters due to its excellent elastomeric properties [12,13,14,15,16,17,18,19,20]. Differing from random P(THF-*co*-CL) as a soft viscous oil obtained by cationic polymerization, multi-block copolymers of [PCL-*b*-P(THF-*co*-CL)]*_m_* are thermoplastic elastomers with high strain at break of over 1000% and moderate tensile strength of 15 MPa, where crystallized PCL segments generate hard domains and amorphous P(THF-*co*-CL) blocks act as soft segments [21]. Such multi-block copolymers can only be obtained via Janus polymerization, which combines living cationic and anionic ROPs at two ends of a polymer chain and finally turns to step-growth polymerization by the coupling of cationic and anionic chain ends [21,22,23].

Nanofibrous structures have a small pore size, a bulky specific surface area and a high sponginess, making them an attractive material for medical applications [7,24].

Electrospinning is a widely accepted technique used to prepare polymer nanofibers, where ultrafine fibers in nanoscale can be collected after an emission of the polymer solution is ejected in an electrical field with high voltage [25,26].

In this paper, the [PCL-*b*-P(THF-*co*-CL)]*_m_* multi-block copolymers obtained via Janus polymerization are used to fabricate an electrospun nanofiber scaffold. The applications of biomedical and biodegradable materials in cardiovascular and other soft tissue engineering could be achieved from these thermoplastic elastomers [21]. The morphology and thermal behavior of electrospun fibers were analyzed using an X-ray diffractometer (XRD), scanning electron microscope (SEM) and differential scanning colorimeter (DSC). It is interesting that the electrospun mat exhibits very different mechanical properties from the bulk material.

## 2. Experimental

### 2.1. Materials

Tetrahydrofuran (THF, Sinopharm Chemical Reagent Co., Shanghai, China) was refluxed over potassium/benzophenone ketyl prior to use. Propylene oxide (PO) was stirred over CaH_2_ and distilled. *ε*-Caprolactone (CL, Sigma Aldrich, St. Louis, MO, USA) was distilled under reduced pressure over CaH_2_ and stored in an argon atmosphere at room temperature. Lu_2_O_3_ (>99.99%) was obtained from Beijing Founde Star Science & Technology Co. (Beijing, China) and used without further purification. Lu(OTf)_3_ was synthesized from Lu_2_O_3_ (Beijing Founde Star Science & Technology Co., Beijing, China) and triflic acid (Energy Chemical Co., Shanghai, China) according to the reported method [19] and dried in vacuum at 200 °C for 40 h. Both [PCL-*b*-P(THF-*co*-CL)]*_m_* multi-block copolymers were synthesized accordingly [21]. Taking **P1** as an example, Lu(OTf)_3_ (0.3662 g, 0.6 mmol) was added into a flame-dried ampoule by Schlenk technique and dissolved in anhydrous THF (4.33 g, 60 mmol) and CL (3.42 g, 30 mmol). PO (50.9 mg, 0.9 mmol) in THF (4.33 g, 60 mmol) was then added to start the polymerization. The ampoule was sealed and kept at 25 °C for 330 h. The crude polymer solution was precipitated in cold methanol, filtrated and dried under vacuum.

### 2.2. Electrospinning

Copolymer [PCL-*b*-P(THF-*co*-CL)]*_m_* 0.25 g was dissolved in 5 mL solvent mixture of dichloromethane and *N,N*-dimethylformamide (3:2 *v*/*v*) and transferred into a 20 mL plastic syringe fitted with a metal needle. Voltage of 8 or 11 kV was applied between the syringe needle and the collector. The distance between the collector and the needle tip was 9 cm. The solution flow rate was 0.5 mL/h. Fibers were collected on a piece of aluminum foil (30 cm × 30 cm). The obtained fiber mat was separated from aluminum foil by immersing in water.

### 2.3. Annealing

Samples of electrospun nanofiber were heated for 8 h at 47 °C and then cooled to room temperature gradually in 25–30 min.

### 2.4. Mechanical Test

The films were cut into dumbbell shapes using a GB/T-528 standard dumbbell cutter. The thicknesses of the samples were measured with a Kafer mechanical thickness gauge (Shanghai Liuling Instrument Factory, Shanghai, China). An electromechanical tensile tester (Reger RWT 10, Shenzhen Reger Instrument Limited Company, Shenzhen, China) instrument was used to perform the macro tensile measurement. All samples were fixed between holders with a distance of 1 cm. Tensile testing was conducted at 10 °C at a rate of 1 mm/min. Tensile strength (in MPa) and strain at break (%) were automatically calculated by the software (RG-Super test).

### 2.5. Characterization

Nuclear magnetic resonance (NMR) spectra were recorded on a Bruker Avance DMX 400 spectrometer (^1^H: 400 MHz) (Billerica, MA, USA) with CDCl_3_ (Sigma-Aldrich, St. Louis, MO, USA) as solvent and tetramethylsilane (TMS) as an internal reference. Molecular weights and polydispersity indices (*Đ*) were determined by size-exclusion chromatography (SEC) on a Waters-150C apparatus equipped with Waters Styragel HR3 and HR4 columns. THF was used as the eluent with a flow rate of 1.0 mL/min at 40 °C, with commercial polystyrene samples as a calibration standard. The morphology of the fiber mats was observed using scanning electron microscope (SEM) (HITACHI S-4800, HITACHI**,** Tokyo, Japan) at an accelerated voltage of 3 kV. Thermal behavior was characterized by differential scanning calorimeter (DSC) (TA Q20, TA Instruments, New Castle, DE, USA) in a temperature range from −90 to 60 °C at a heating rate of 10 °C/min. Crystal structure of copolymer was investigated using X-ray diffraction (XRD) on an X’Pert PRO (Panalytical Inc., Almelo, The Netherlands) equipment with a CuKα source.

## 3. Results and Discussion

**Scheme 1:**
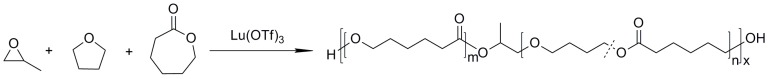
Janus polymerization of CL and THF catalyzed by Lu(OTf)_3_/PO.

Two [PCL-*b*-P(THF-*co*-CL)]*_m_* samples were synthesized by Janus polymerization of CL with THF according to our previous report as shown in Scheme 1 [21]. Their chemical compositions were adjusted by varying the monomer ratio of CL and THF, and characterized by ^1^H NMR and SEC, with the results listed in Table 1. In brief, a mixture of CL, THF, lutetium triflate and propylene oxide was allowed to polymerize for 2 weeks. The proportion of CL in final polymers can be tuned by adjusting the feed ratio of CL and THF. **P1** and **P2** contain 53.3 and 88.4 wt % of CL, respectively, according to ^1^H NMR analyses (Figure S1 in Supplementary Materials). Their number-average molecular masses were 97 and 131 kg/mol, respectively, according to SEC. Their polydispersity was close to 2.0 due to step-growth polymerization in the third-stage of Janus polymerization [21].

**Figure 1:**
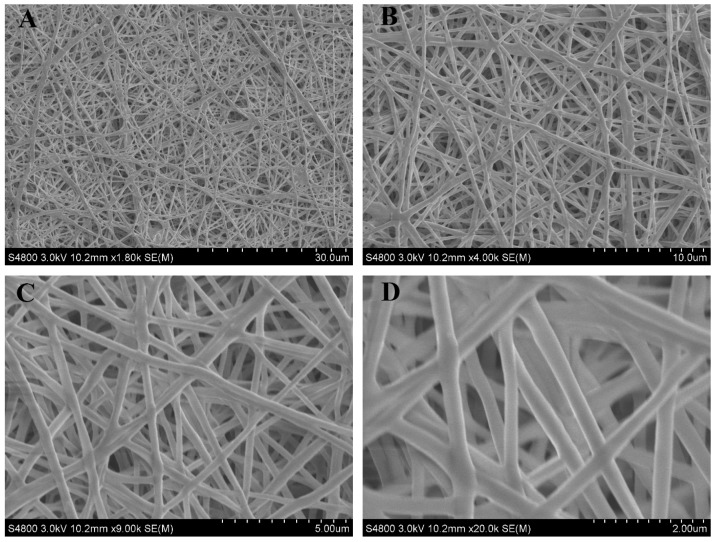
SEM images of the electrospun fibers of **P1** (**A**,**C**) and **P2** (**B**,**D**) show random orientation.

Nanofibers of the two samples were produced by electrospinning. Their SEM images (Figure 1) clearly illustrated randomly oriented fibers without obvious beads. They were cross-linked with each other during the drying process and formed a non-woven mat. The fibers’ diameters measured by SEM ranged from 290 to 520 nm. The porous structure could be useful in biomedical applications, for instance, for cell incubation. A macroscale photograph is also shown in Figure S2 in Supplementary Materials.

**Figure 2:**
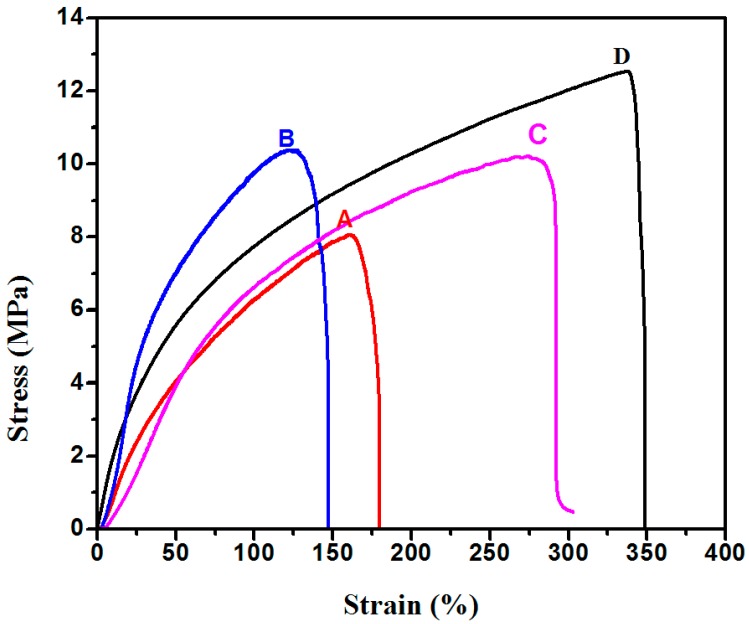
Stress-strain curves of the electrospun mats of **P1** (**A** in red), **P2** (**B** in blue), and **P1** annealed (**C** in magenta) and **P2** annealed (**D** in black) at 47 °C.

The fibers’ scaffolds, in dumbbell shapes, were cut and fixed in the tensile tester and stretched in order to determine their macroscopic mechanical properties. The results are summarized in Table 1 and Figure 2. **P1** and **P2** electrospun mats exhibited a tensile strength of 8.1 and 10.4 MPa, respectively. **P2** with higher CL percentage was slightly stronger than **P1**. They were both white and flexible plastic films with less elasticity, with strains at break between 123% and 161%, respectively. This observation differs very much from the corresponding thermal-molded films of the same polymers, which had excellent elasticity, with strain at break of 2610% and 1020%, respectively (Table 1). After electrospinning, the multi-block copolymers behaved like plastic.

**Figure 3:**
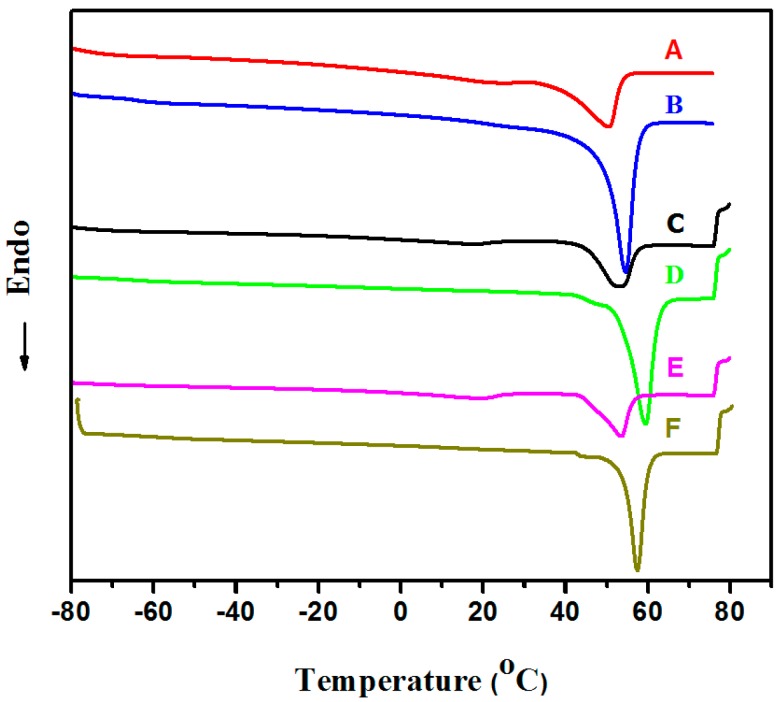
DSC curves of the electrospun mats of **P1** (**A** in red) and **P2** (**B** in blue), **P1** annealed (**C** in black) and **P2** annealed (**D** in green**)** after annealing at 47 °C, P1 cast film (**E** in magenta) and P2 cast film (**F** in dark yellow).

**Figure 4:**
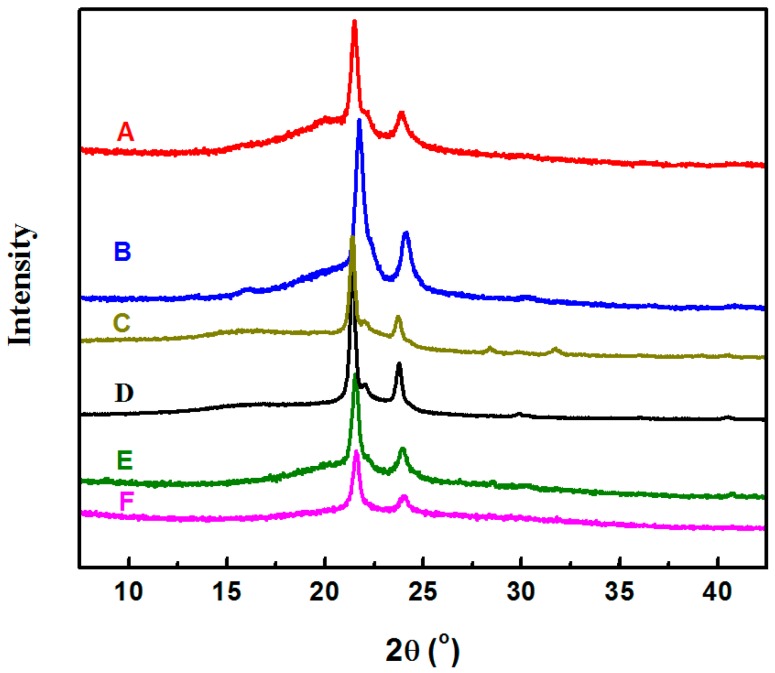
XRD patterns of the electrospun mats of **P1** (**A** in red) and **P2** (**B** in blue), **P1** annealed (**C** in dark yellow) and **P2** annealed (**D** in black) at 47 °C, casted films of **P1** (**E** in olive) and **P2** (**F** in magenta). Crystallinity is determined from ratio of the area under the peaks to the total area under the curve as reported in the literature [27].

In order to measure the crystallinity of PCL blocks in electrospun mats, we checked their thermal behavior by DSC measurements and their crystallinity by XRD analyses. According to the DSC curves in Figure 3, the electrospun mats of **P1** and **P2** had almost the same melting temperatures, at 51.0 and 54.0 °C, respectively, which is close to the reported data (55.0 °C) of the corresponding thermal-molded films [21]. Their XRD patterns support the idea that the crystals belong to PCL blocks, with sharp peaks at 21.6 °C and 24.0 °C, as shown in Figure 4. The crystallization degrees of **P1** and **P2** electrospun mats were 17.4% and 22.5%, respectively, which was higher than those of the corresponding thermal-molded films (5.7% and 7.1%, respectively) in XRD. The same tendency was also found in DSC (Table 1), but wasn’t as pronounced. It is understandable that the PCL segments in the polymer are more stretched during the electrospinning process, increasing their orientation. The degree of crystallization of **P2** with a higher PCL fraction and longer PCL chains was higher than that of **P1** after both of the processing methods. Larger crystallization domains are expected during the fast drying process in electrospinning and frozen in nanofibers together with amorphous segments. However, the difference between the degree of crystallization of electrospun nanofiber and thermal-molded film is not sufficient to explain the higher difference of the mechanical properties.

**Figure 5:**
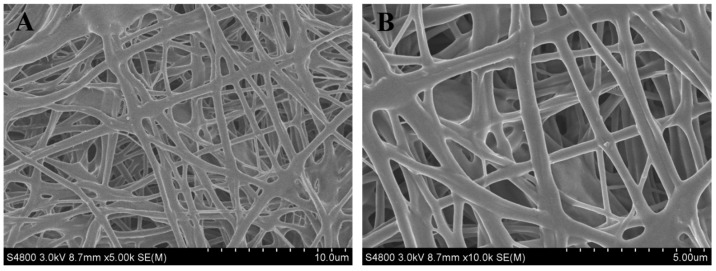
SEM images of electrospun nanofibers of **P1** (**A**) and **P2** (**B**) after annealing at 47 °C.

This could be another reason that phase separations of the crystal hard domain of PCL blocks and the soft domain of P(THF-*co*-CL) didn’t have enough time to complete during the fast electrospinning process [23]. Annealing was a suitable way to increase the completion of phase separation by activating the frozen entities of polymer backbones. We put the electrospun mat of **P1** and **P2** in an oven and kept it at 47 °C for 8 h. The nanofibers were well maintained when the annealing temperature was lower than its melting temperature (Figure 5). According to the DSC measurements, annealing increased the crystallinity of PCL in the electrospun mat, giving a higher *T*_m_ of 60 °C, the same as the value of homopolymer of PCL [28]. XRD analysis illustrates an increased degree of crystallization up to 31.2%, which was much higher than that of thermal-molded film of **P2**. After annealing, both tensile strength and strain at break of **P2** increased by 2.1 MPa and 215%, respectively (Table 1). The annealing process improved the mechanical properties of the electrospun mat as the nanofibers are more elastic and stronger. However, they were still lower than those of the corresponding thermal-molded films. Therefore, crystallinity alone cannot explain the decrease of the electrospun mat in both the tensile strength and strain at break.

**Figure 6:**
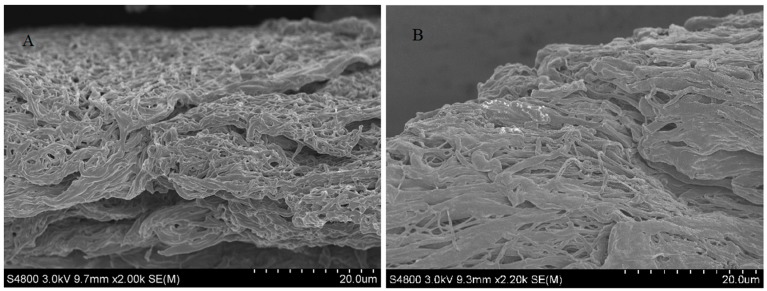
SEM images of the broken edge of the electrospun nanofibers mat of **P1** (A) and **P2** (B) after stretching in mechanical test.

There is insufficient proof to conclude that crystallinity or phase separation is responsible for the loss of elasticity. Unlike the thermal-molded films, the electrospun fibers may have higher crystallinity, but the interaction between fibers could be a weak point when stretching. Annealing partially enhances the interaction among fibers due to potential enhancement of fusion between fibers at merging points. We analyzed the electrospun mat after being broken in a mechanical test by SEM (Figure 6), and didn’t find broken fibers, but only saw relaxed fibers at the edge. It is reasonable that the junction points between nanofibers are the weakest positions during stretching.

## 4. Conclusions

Multi-block copolymers of [PCL-*b*-P(THF-*co*-CL)]*_m_* obtained by Janus polymerization were successfully electrospun into nanofibers with diameters of between 290 and 520 nm. The white electrospun mats exhibited plastic properties with a tensile strength of 10.4 MPa and strain at break of 123%. Though the mechanical properties improved slightly after annealing, they were very different from the corresponding thermal-molded films with the same copolymers, which exhibited elasticity with strain at break up to 2610%. This is a result of the weak conjunctions amongst nanofibers, which were easy to break during stretching. The electrospinning process results in materials different from thermal-molded ones, and both are promising materials with their own properties.

## Figures and Tables

**Table 1:** Multi-block copolymers and their properties.

Sample	CL Content (wt %) ^a^	*M*_n_^b^ (kg/mol)	*Đ* ^b^	*T*_m_ (°C)	Δ*H* (J/g)	*χ*_DSC_ (%)	*χ*_XRD_ (%)	Tensile Strength (MPa)	Strain at Break (%)
**P1**-nanofiber	53.3	97	2.09	51.0	32.64	43	17.4	8.1	161
**P1**-annealed				53.2	24.45	32	21.1	10.2	267
**P1**-cast film				53.5	26.60	35	17.4	-	-
**P1**-thermal-molded film				50.0	21.18	28	5.7	20.0	2610
**P2**-nanofiber	88.4	131	2.06	54.0	40.57	32	22.5	10.4	123
**P2**-annealed				60.0	50.80	41	31.2	12.5	338
**P2**-cast film				55.5	43.06	35	19.3	-	-
**P2**-thermal-molded film				55.0	37.09	30	7.1	17.1	1020

^a^ Calculated from ^1^H NMR. ^b^ Determined by SEC.

## Supplementary Materials

The following are available online at www.mdpi.com/2073-4360/9/11/559/s1.
