# Correction: Shah, M.I., et al. Properties of Electrospun Nanofibers of Multi-Block Copolymers of [Poly-ε-Caprolactone-b-Poly(tetrahydrofuran-*co*-ε-caprolactone)]m Synthesized by Janus Polymerization. *Polymers* 2017, *9*, 559

**DOI:** 10.3390/polym13010111

**Published:** 2020-12-29

**Authors:** Muhammad Ijaz Shah, Zhening Yang, Yao Li, Liming Jiang, Jun Ling

**Affiliations:** MOE Key Laboratory of Macromolecular Synthesis and Functionalization, Department of Polymer Science and Engineering, Zhejiang University, Hangzhou 310027, China; muhammadijazshas@yahoo.com (M.I.S.); yznfly@zju.edu.cn (Z.Y.); 21429011@zju.edu.cn (Y.L.); cejlm@zju.edu.cn (L.J.)

The authors wish to make the following changes to the published paper as listed below [1]. In the original manuscript, there are some mistakes in captions of Figures 1, 5 and 6; the call out of Figure 1,5 and two units in the sixth line of Page 5. The corrected sentences are shown below:

The Caption of Figure 1 should be: Figure 1. SEM images of the electrospun fibers of P1 (A) 30 µm; (B) 10 µm; (C) 5 µm and (D) 2 µm.

The Caption of Figure 5 should be: Figure 5. SEM images of the electrospun fibers of P2 after annealing at 47 °C (A) 10 µm and (B) 5 µm.

The Caption of Figure 6 should be: Figure 6. SEM images of the broken edge of the electrospun nanofibers mat after stretching in mechanical test.

Besides of these, the first line below Table 1 in Page 3: “Their SEM images (Figure 1)” is a little bit confusing. The correct text is “The SEM images of P1 (Figure 1)”.

The sixth line in Page 5: the unit in “at 21.6 °C and 24.0 °C” is incorrect. The correct text is “at 21.6° and 24.0°”.

The 6th line in Page 6: “(Figure 5)” is not accurate. The correct text is “(P2 as an example shown in Figure 5)”.

The authors apologize for any inconvenience caused, and the changes do not affect the scientific results. The manuscript will be updated, and the original will remain online on the article webpage at https://www.mdpi.com/2073-4360/9/11/559.
