# Supplementary material for: Properties of Electrospun Nanofibers of Multi-Block Copolymers of [Poly-ε-caprolactone-b-poly(tetrahydrofuran-co-ε-caprolactone)]m Synthesized by Janus Polymerization
Source: Polymers (Basel). 2017 Oct 27;9(11):559. doi: 10.3390/polym9110559 (PMC6418973; doi:10.3390/polym9110559)
Supplement: Supplementary file 1 [file polymers-09-00559-s001.pdf]

Properties of electrospun nano-fibers of multi-block copolymers of [poly-ε-caprolactone-*b*-poly(tetrahydrofuran-*co*-ε-caprolactone)]$_m$ synthesized by Janus polymerization

Muhammad Ijaz Shah, Zhening Yang, Yao Li, Liming Jiang, Jun Ling*

MOE Key Laboratory of Macromolecular Synthesis and Functionalization, Department of Polymer Science and Engineering, Zhejiang University, Hangzhou 310027, China.

* Corresponding author. Email: lingjun@zju.edu.cn

Supporting Information

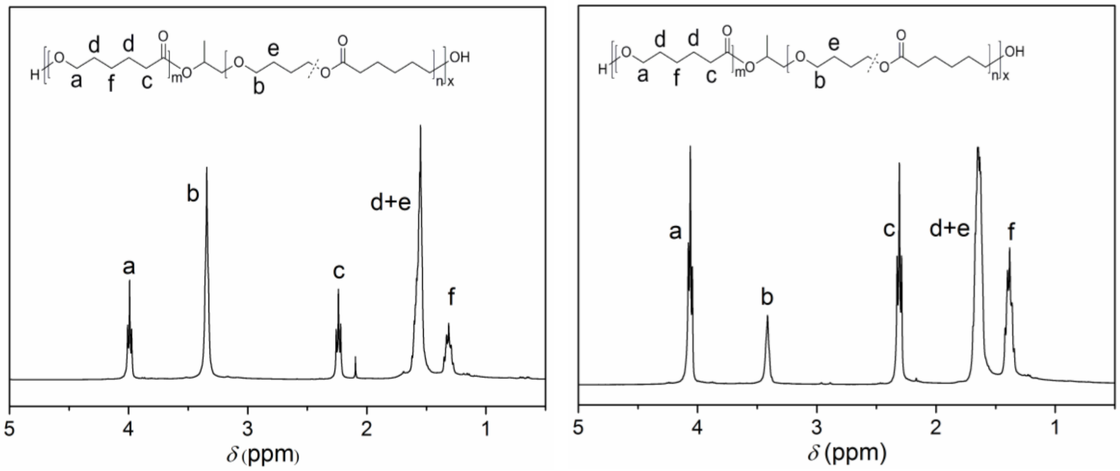

Figure S1. ^{1}H NMR spectra of **P1** (left) and **P2** (right)

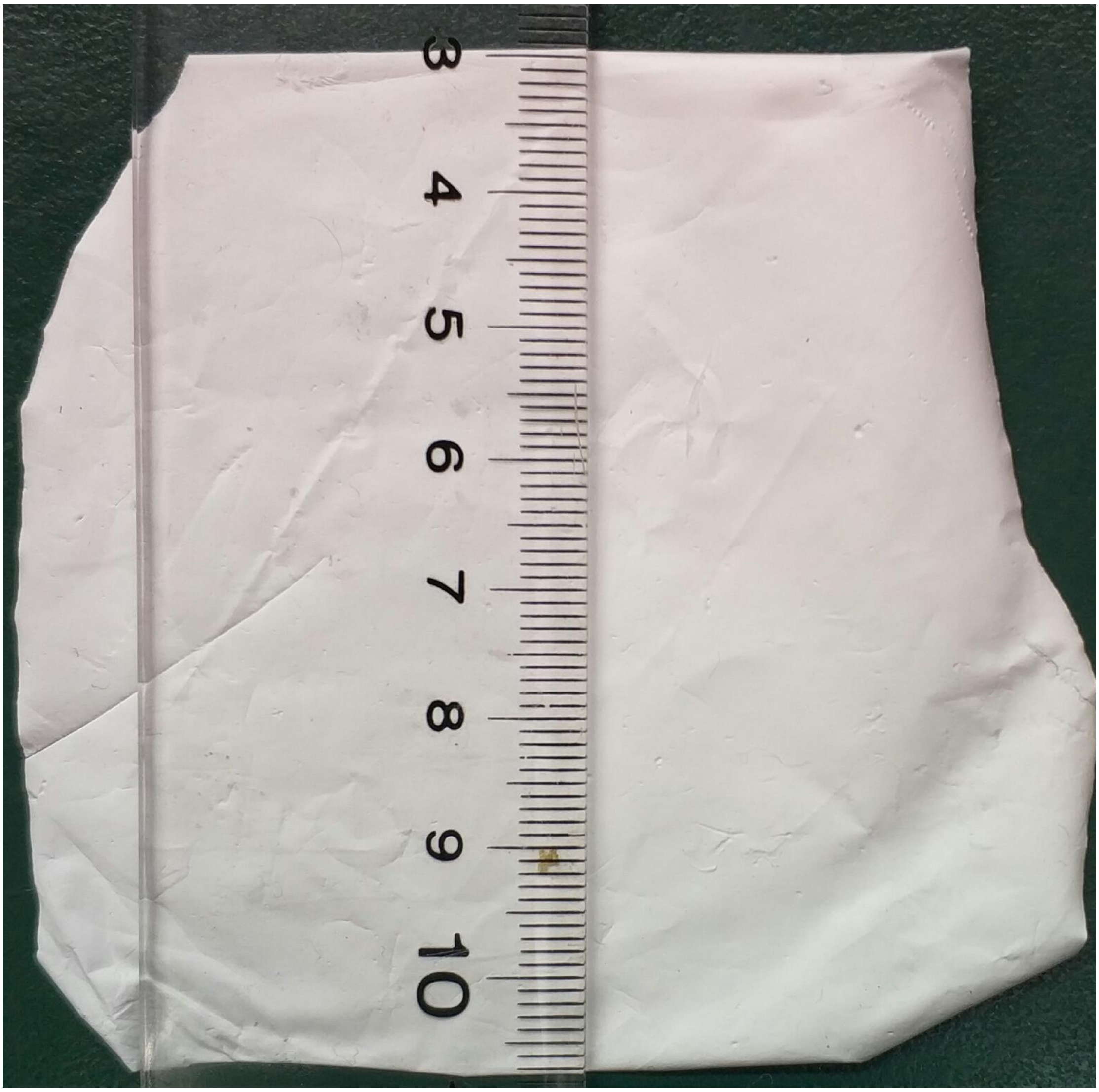

Figure S2. Macroscale image of electrospun nanofibers mat
